# Anti-Angiogenic Potential of Marine *Streptomyces*-Derived Lucknolide A on VEGF/VEGFR2 Signaling in Human Endothelial Cells

**DOI:** 10.3390/molecules30050987

**Published:** 2025-02-20

**Authors:** Byeoung-Kyu Choi, Min-Hee Jo, Hee Jae Shin, Sun Joo Park

**Affiliations:** 1 Department of Bio-Convergence Engineering, Dongyang Mirae University, Seoul 08221, Republic of Korea; choibk4404@dongyang.ac.kr; 2 BB21 Plus Program, Department of Chemistry, Pukyong National University, Busan 48513, Republic of Korea; myhappy5951@naver.com; 3 Marine Natural Products Laboratory, Korea Institute of Ocean Science and Technology, Busan 49111, Republic of Korea; 4 Department of Marine Technology and Convergence Engineering, Korea National University of Science and Technology (UST), Daejeon 34113, Republic of Korea

**Keywords:** Lucknolide A, marine natural compound, marine *Streptomyces*, anti-angiogenesis, VEGF/VEGFR2 signaling, anti-tumor

## Abstract

Angiogenesis, primarily driven by the vascular endothelial growth factor (VEGF) and its receptor, the VEGFR, plays a key role in various pathological processes such as cancer progression. Here, we investigated the anti-angiogenic effects of Lucknolide A (LA), a marine *Streptomyces*-derived compound, and evaluated its potential as a VEGFR2 inhibitor. LA selectively inhibited the proliferation of human endothelial cells EA.hy926 and HUVEC while exhibiting minimal effects on normal fibroblasts and various tumor cells. LA induced S-phase cell cycle arrest and apoptosis in EA.hy926 cells, increasing apoptotic markers p53, Bax, and p21 and decreasing the anti-apoptotic protein Bcl-2, with these effects being further enhanced under VEGF stimulation. Additionally, LA suppressed VEGFR2 phosphorylation and its downstream signaling pathways, including Akt/mTOR/p70S6K, MEK/ERK, Src, FAK, and p38 MAPK, which are crucial for endothelial survival and angiogenesis. Molecular docking studies revealed that LA binds to both inactive (DFG-out, PDB: 4ASD) and active (DFG-in, PDB: 3B8R) VEGFR2 conformations, with a significantly stronger affinity for the active state (−107.96 kcal/mol) than the inactive state (−33.56 kcal/mol), suggesting its potential as a VEGFR2 kinase inhibitor. Functionally, LA significantly inhibited VEGF-induced endothelial migration, tube formation, and microvessel sprouting in both in vitro and ex vivo rat aortic ring assays. Additionally, LA reduced tumor-associated tube formation induced by human breast tumor cells (MDA-MB-231), indicating its potential to suppress VEGF-dependent tumor angiogenesis. These findings suggest that LA is a promising selective anti-angiogenic agent with potential therapeutic applications in angiogenesis-related diseases such as cancer.

## 1. Introduction

Angiogenesis, the formation of new blood vessels, plays a crucial role in various physiological and pathological processes including tissue growth and repair, vascular diseases, and cancer progression [1,2]. This process is highly complex and is primarily regulated by the vascular endothelial growth factor (VEGF), a key pro-angiogenic molecule that binds to its receptors, VEGF receptors (VEGFRs), on endothelial cells. Upon binding, the VEGF activates downstream signaling pathways such as mitogen-activated protein kinases (MAPKs) and Akt, which promote endothelial cell growth, survival, migration, and tube formation [2,3].

Tumor-induced angiogenesis is a hallmark of cancer, and the inhibition of angiogenesis is a viable therapeutic strategy to suppress tumor growth and metastasis [4]. Several anti-angiogenic agents targeting the VEGF signaling pathway, including the anti-VEGF antibody bevacizumab (Avastin^®^, Genentech, South San Francisco, CA, USA) and tyrosine kinase inhibitors sorafenib (Nexavar^®^, Bayer, Leverkusen, Germany) and sunitinib (Sutent^®^, Pfizer, New York, NY, USA), have been approved for cancer treatment [5]. However, these agents have significant clinical limitations, such as severe side effects, the development of resistance, and limited efficacy in certain cancer types. These limitations are primarily due to their low specificity and broad multi-target inhibition, which affect various cell types in the body [5,6]. Therefore, there is a growing need for more selective anti-angiogenic and anti-proliferative agents that specifically target endothelial cells. Although these angiogenesis inhibitors may not directly kill cancer cells, they can effectively inhibit tumor growth and metastasis and can be administered over an extended period [7,8].

Marine organisms have gained attention as potential sources of novel secondary metabolites with anti-angiogenic properties [9,10]. The vast biodiversity and chemical diversity of marine ecosystems provide rich resources for the discovery of new bioactive compounds. Several marine-derived compounds, such as bryostatin-1 [10], plitidepsin [11], eribulin mesylate [12], and marizomib [10], have demonstrated anti-angiogenic activity and have progressed to clinical trials for cancer therapy. However, issues regarding the toxicity and efficacy of these drugs remain unresolved [13]. Consequently, the development of anti-angiogenic agents from marine sources is still in its early stages.

As part of our ongoing research on marine anti-angiogenic agents, we recently isolated Lucknolide A (LA) from a marine-derived *Streptomyces* sp. strain 151KO-065 [14]. LA had previously been isolated from a terrestrial actinomycete, but its biological activity has not been reported [15]. Consistent with this, our previous experiments using several tumor cell lines, including B16F10, A375, HeLa, HepG2, MDA-MB231, and PC3, also did not reveal any anti-tumor activity of LA [14]. Thus, the biological activity and therapeutic potential of LA remain largely unexplored.

In this study, we found that LA selectively inhibited the proliferation of endothelial cells compared to that of other cell types, including tumor cells. Treatment with LA induced anti-angiogenic effects by promoting cell cycle arrest and apoptosis and inhibiting endothelial cell migration, tube formation, and microvessel sprouting. These effects are associated with the suppression of the VEGF/VEGFR2 signaling pathway, a key regulator of angiogenesis. Molecular docking studies further revealed that LA preferentially binds to the active VEGFR2 conformation (DFG-in) with significantly higher affinity (−107.96 kcal/mol) compared to the inactive state (−33.56 kcal/mol), suggesting its potential role as a selective VEGFR2 inhibitor. Additionally, LA suppressed tube formation stimulated by MDA-MB-231 human breast tumor cells, suggesting its potential to inhibit tumor-induced angiogenesis.

## 2. Results

### 2.1. Isolation of Lucknolide A (LA)

**Figure 1:**
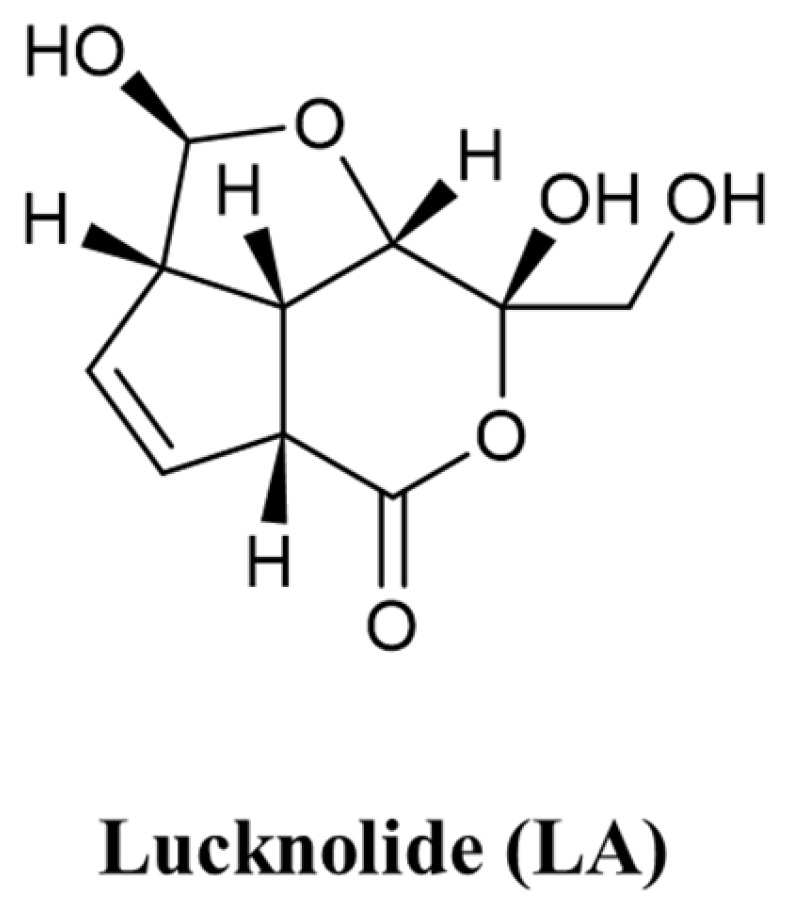
The structure of Lucknolide A (LA). LA was isolated from a marine-derived *Streptomyces* sp. strain 151KO-065.

Lucknolide A (LA) was isolated and structurally identified from a marine-derived *Streptomyces* strain 151KO-065, as described in our recent study [14]. The strain 151KO-065 was isolated from a mangrove sample collected near Kosrae Island and identified as *Streptomyces* sp. by 16S rRNA gene sequence analysis. After large-scale fermentation, the culture broth was subjected to solvent extraction, and the culture extract was fractionated using column chromatography. A fraction with unusual peaks in the ^1^H NMR spectrum was further purified using HPLC, leading to the isolation of LA. LA was obtained as a white powder and displayed an [M−H]^−^ ion peak at *m*/*z* 227.17, corresponding to a molecular formula of C_10_H_12_O_6_. Analysis of ^1^H, ^13^C NMR, HSQC, ^1^H-^1^H COSY, and HMBC spectra confirmed the structure of LA (Figure 1).

LA was initially isolated from a terrestrial actinomycete, but no biological activities have been reported to date [15]. In our previous study on the anti-tumor activity of LA from marine *Streptomyces* against various tumor cell lines, no significant results were observed [14]. Consequently, the biological properties of LA remain largely unexplored. To address this, we expanded the scope of our research to investigate the effects of LA on endothelial cells. This study aimed to elucidate the anti-angiogenic effects of LA and investigate its underlying molecular mechanisms.

### 2.2. LA Decreases the Proliferation of Human Endothelial Cells

**Figure 2:**
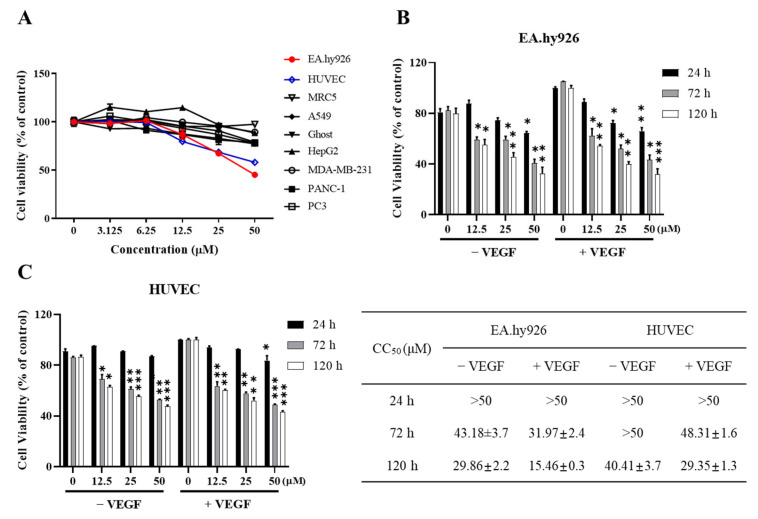
LA decreases the proliferation of EA.hy926 and HUVEC cells in a dose- and time-dependent manner. (**A**) EA.hy926, HUVEC, MRC5, A549, Ghost, HepG2, MDA-MB-231, PANC-1, and PC3 cells were treated with various concentrations (0–50 μM) of LA for 72 h. Cell viability was determined by MTT assay. Each value is expressed as the mean ± standard deviation (SD) of three independent experiments. (**B**) EA.hy926 and (**C**) HUVEC cells were treated with 0–50 µM LA in the presence or absence of 50 ng/mL of the VEGF for different time intervals (24 h, 72 h, and 120 h). CC_50_ (μM) is expressed as the mean ± standard deviation (SD) of three independent experiments. Statistical significance is indicated as * *p* < 0.05, ** *p* < 0.01, and *** *p* < 0.001. LA-only treated groups were compared to the vehicle control (DMSO), while LA + VEGF-treated groups were compared to the VEGF.

We first examined whether LA inhibits the proliferation of endothelial cells. Human umbilical vein endothelial EA.hy926 and HUVEC cells were treated with various concentrations of LA, and cell viability was analyzed over time. Notably, LA treatment decreased endothelial cell proliferation in dose- and time-dependent manners (Figure 2A). Moreover, LA had minimal effects on other human cell types, including normal fibroblasts (MRC5) and several tumor cell lines (A549, Ghost, HepG2, MDA-MB-231, PANC-1, and PC3), suggesting that LA selectively inhibits the growth of endothelial cells.

Furthermore, the inhibitory potential of LA on endothelial cells increased with longer treatment duration, particularly in the presence of the VEGF (Figure 2B). The CC_50_ values of LA after 120 h of treatment were 29.86 μM in the absence of the VEGF and 15.46 μM in the presence of the VEGF for EA.hy926 cells, and 40.41 μM in the absence of the VEGF and 29.35 μM in the presence of the VEGF for HUVEC cells. These results suggest that LA is more effective in the presence of the VEGF during extended treatment periods in both endothelial cell types.

### 2.3. LA Causes S-Phase Cell Cycle Arrest and Apoptosis of EA.hy926 Endothelial Cells

**Figure 3:**
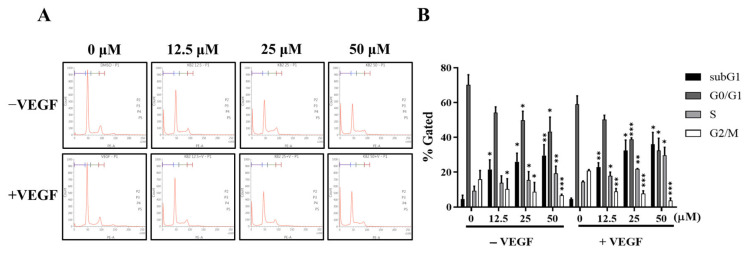
LA causes the S-phase cell cycle arrest of EA.hy926 cells in a dose-dependent manner. (**A**) Representative cell cycle analysis images showing LA-induced S-phase arrest. EA.hy926 cells were treated with 0, 12.5, 25, and 50 μM LA for 72 h in the presence or absence of 50 ng/mL of the VEGF. Cells were fixed in ethanol and stained with propidium iodide. Cells were analyzed using flow cytometry with single histogram statistics. (**B**) Data are expressed as the mean ± SD of three independent experiments. Statistical significance is indicated as * *p* < 0.05, ** *p* < 0.01, and *** *p* < 0.001. LA-only treated groups were compared to the vehicle control (DMSO), while LA + VEGF-treated groups were compared to the VEGF-treated condition.

To further explore how LA inhibited endothelial cell proliferation, we analyzed its impact on cell cycle progression in EA.hy926 cells. As shown in Figure 3, LA treatment significantly increased the proportion of cells in the Sub G1 and S-phases in a dose-dependent manner, while reducing the percentage of cells in the G0/G1 and G2/M phases. After 72 h, the S-phase percentage in EA.hy926 cells increased from 9.45% in the control to 13.7%, 15.4% (* *p* < 0.05), and 19.3% (** *p* < 0.01) in cells treated with 12.5, 25, and 50 μM of LA, respectively. VEGF co-treatment further increased the percentage of cells in the S-phase to 17.9% (* *p* < 0.05), 21.1% (** *p* < 0.01), and 29.8% * (*p* < 0.05), respectively.

Additionally, the percentage of cells in the Sub G1 phase, indicating apoptotic cell death, increased from 4.5% in the control to 28.5% (** *p* < 0.01) with LA treatment, and to 36.1% (* *p* < 0.05) with LA and the VEGF in combination. These results suggest that LA induces S-phase cell cycle arrest, leading to apoptotic cell death, and that VEGF co-treatment further enhances the effect of LA.

**Figure 4:**
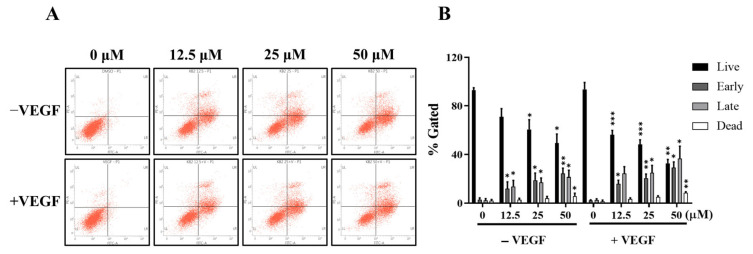
LA induces the apoptosis of EA.hy926 cells in a dose-dependent manner. (**A**) Representative images showing LA-induced apoptosis. EA.hy926 cells were treated with 0, 12.5, 25, and 50 μM LA for 72 h in the presence or absence of 50 ng/mL of the VEGF and analyzed by Annexin V-PI staining. (**B**) Cells that appeared Annexin V-positive/PI-negative (**lower right**) are considered early-apoptotic cells, while Annexin V/PI double-positive (**upper right**) are considered late-apoptotic cells. The percentage of live (**lower left**), early apoptotic, late-apoptotic, and dead (**upper left**) cells. Data are expressed as the mean ± SD of triplicate independent experiments. Statistical significance is indicated as * *p* < 0.05, ** *p* < 0.01, and *** *p* < 0.001. LA-only treated groups were compared to the vehicle control (DMSO), while LA + VEGF-treated groups were compared to the VEGF-treated condition.

To further confirm the apoptotic effect of LA, flow cytometric analysis using Annexin V/Propidium Iodide staining was performed (Figure 4). As shown by fluorescence-activated cell sorting (FACS) data, LA treatment increased the population of apoptotic cells in a dose-dependent manner. Treatment with 50 μM of LA increased early apoptosis (24.5%) and late apoptosis (21.4%) by more than 8-fold compared to the control cells (2.9% and 2.2%, respectively). In combination with the VEGF, LA further enhanced apoptosis, increasing early and late apoptotic cell populations by 29.2% * (*p* < 0.05) and 36.5% * (*p* < 0.05), respectively. These results are consistent with the synergistic effect of LA and the VEGF in increasing the Sub G1 cell fraction, as shown in Figure 3, suggesting that VEGF stimulation increases the sensitivity of endothelial cells to LA, leading to enhanced cell cycle arrest and apoptosis.

**Figure 5:**
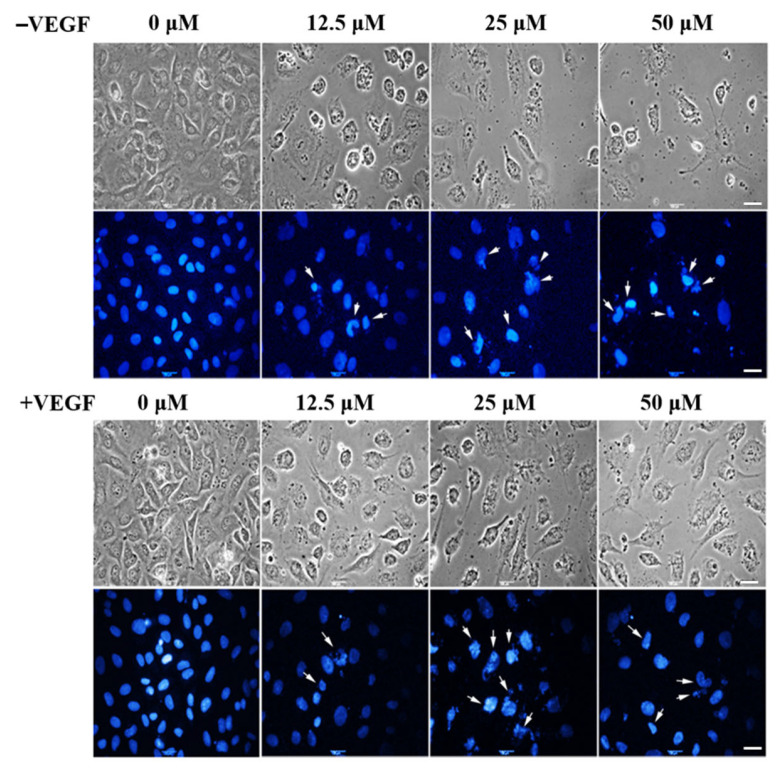
The effect of LA on the nuclei morphology of EA.hy926 cells by Hoechst 33,258 staining. EA.hy926 cells were cultured with 0, 12.5, 25, and 50 μM of LA for 72 h in the presence or absence of 50 ng/mL of the VEGF. The nuclei were visualized under an inverted fluorescent microscope (40×). Arrows indicate apoptotic nuclei. Scale bar, 100 μm.

LA also induced nuclear morphological changes in endothelial cells (Figure 5). After exposure to LA, apoptotic cells displaying abnormal nuclear shrinkage, chromatin condensation, and fragmentation were observed by Hoechst staining both in the presence and absence of the VEGF (indicated by arrows in Figure 5). Collectively, these data indicate that LA induces S-phase cell cycle arrest and triggers typical apoptotic cell death in endothelial EA.hy926 cells.

### 2.4. LA Suppresses the Activation of VEGFR2 and Its Downstream Signaling Pathway

**Figure 6:**
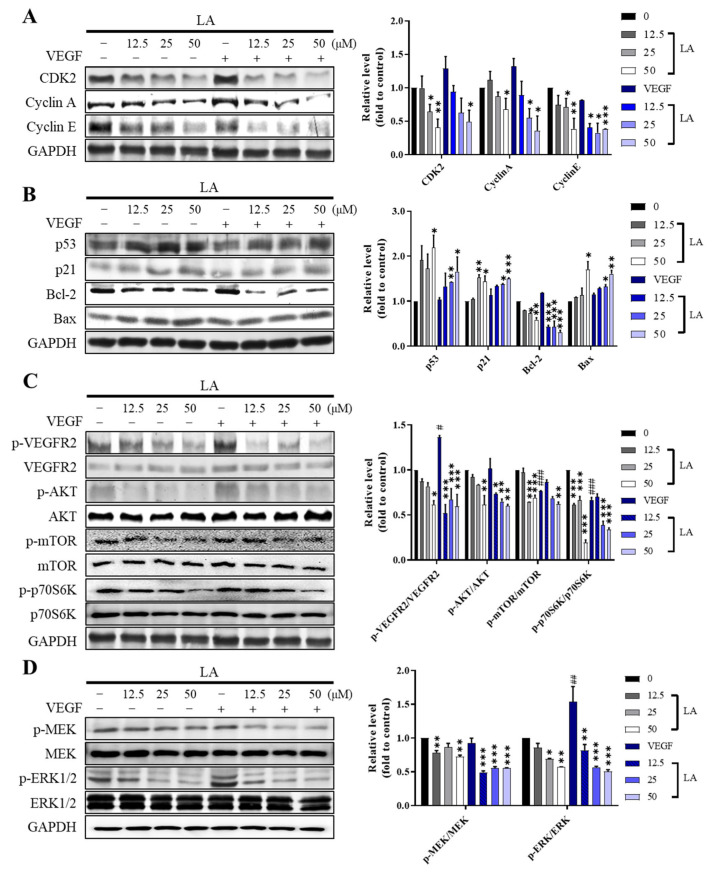

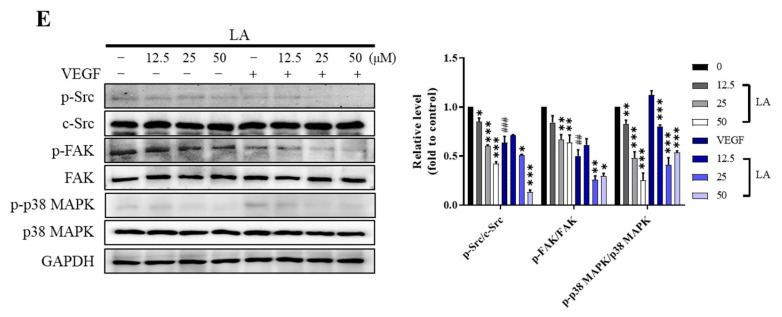
The effect of LA on the proteins involved in S-phase cell cycle arrest and apoptosis in EA.hy926 cells. Representative western blot images indicating LA-induced S-phase arrest (**A**) and apoptosis (**B**). EA.hy926 cells were treated with 0, 12.5, 25, and 50 µM LA for 72 h in the presence or absence of 50 ng/mL of the VEGF. * *p* < 0.05, ** *p* < 0.01, and *** *p* < 0.001, compared to vehicle DMSO control. (**C**) LA suppresses the phosphorylation of VEGFR2 and its downstream effectors, (**C**) Akt/mTOR/p70S6K (**D**) MEK/ERK (**E**) Src, FAK, and p38 MAPK. The results were quantified using ImageJ (v1.54f) and are presented as the mean ± SD of three independent experiments. Statistical significance is indicated as * *p* < 0.05, ** *p* < 0.01, and *** *p* < 0.001. LA-only treated groups were compared to the vehicle control (DMSO), while LA + VEGF-treated groups were compared to the VEGF-treated condition. # *p* < 0.05, ## *p* < 0.01, and ### *p* < 0.001, compared to the DMSO control.

To investigate the molecular mechanisms underlying LA-induced cell cycle arrest and apoptosis, we examined the expression and activation of key proteins involved in cell cycle regulation and apoptosis (Figure 6). LA treatment decreased the expression of cyclins A and E and CDK2, which are essential regulators of S-phase progression (Figure 6A). The combination of LA and the VEGF further reduced the expression of these proteins compared to LA alone. LA also dose-dependently increased the expression of p53, a key regulator of cell cycle arrest and apoptosis, and the proapoptotic proteins p21 and Bax, while decreasing the expression of the anti-apoptotic protein Bcl-2 (Figure 6B). These findings suggest that the antiproliferative effect of LA is mediated by the downregulation of anti-apoptotic signaling involving Bcl-2, as well as by the upregulation of proapoptotic signaling through the Bax and p53 pathways.

Since endothelial cell proliferation and angiogenesis are primarily driven by the VEGF/VEGFR2 signaling pathway, and the effect of LA was more pronounced in cells co-treated with the VEGF, we next examined the phosphorylation of VEGFR2 and its downstream effectors (Akt/mTOR/p70S6K, MEK/Erk, SRC, FAK, and p38) (Figure 6C–E). LA significantly reduced the phosphorylation levels of VEGFR2 and its downstream signaling in a dose-dependent manner, and VEGF co-treatment further enhanced the inhibitory effects of LA, indicating a suppressive effect of LA on the VEGF/VEGFR2 signaling pathway.

### 2.5. LA Inhibits the VEGF-Induced Cell Migration and Tube Formation of EA.hy926 Cells

Endothelial cell migration and tube formation are essential steps of angiogenesis [16,17,18]. To determine whether LA affects VEGF-induced endothelial cell migration and tube formation, we performed a wound-healing assay and a two-dimensional Matrigel tube formation assay on EA.hy926 cells in the presence or absence of the VEGF.

**Figure 7:**
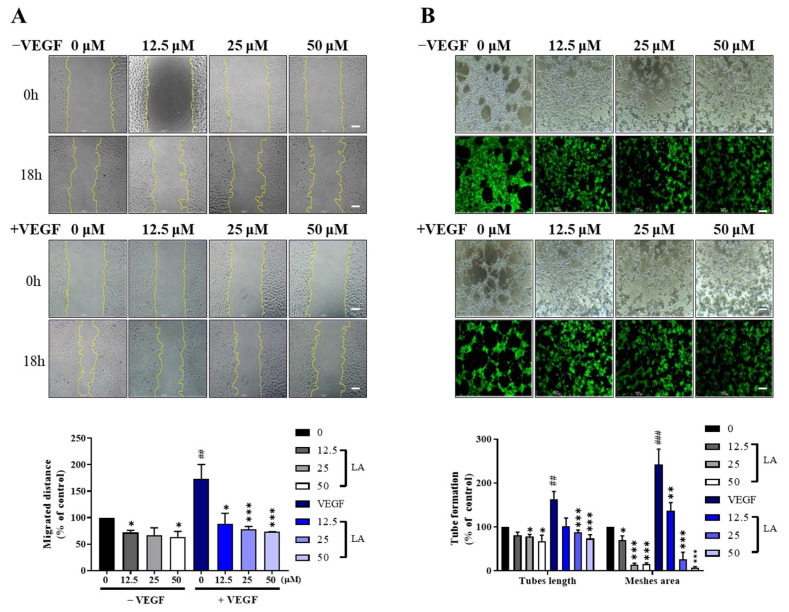
LA inhibits the VEGF-induced migration and tube formation of EA.hy926 cells. (**A**) Wound-healing scratch assays were performed on EA.hy926 cells plated onto Matrigel-coated dishes. After serum starvation, cells were pre-cultured with or without various concentrations of LA for 48 h and then further incubated with LA for 12 h in the presence or absence of 50 ng/mL of the VEGF. A sterile 200 μL pipette tip was used to create a scratch wound. Cell migration was quantified by measuring the gap size in 4 different images, and representative images are shown. Data are expressed as mean ± SD from three independent experiments. (**B**) EA.hy926 cells were pre-treated with 0, 12.5, 25, and 50 µM LA for 48 h and then further incubated with LA for 12 h in the presence or absence of 50 ng/mL of the VEGF. Tube formation was quantified by measuring the average of 4 different images, and representative images are shown. Data are expressed as the mean ± SD of three independent experiments. Statistical significance is indicated as * *p* < 0.05, ** *p* < 0.01, and *** *p* < 0.001. LA-only treated groups were compared to the vehicle control (DMSO), while LA + VEGF-treated groups were compared to the VEGF. ## *p* < 0.01, and ### *p* < 0.001, compared to the DMSO control. Scale bar, 100 μm.

LA inhibited the migration and tube formation of EA.hy926 cells in a dose-dependent manner and VEGF stimulation further enhanced these effects of LA (Figure 7A,B). In additional experiments using HUVECs, consistent with our results in EA.hy926 cells, LA suppressed VEGF-induced tube formation in a dose-dependent manner, with VEGF stimulation further enhancing its inhibitory effect (Supplementary Figure S1). These findings clearly demonstrate that LA inhibits endothelial cell migration and tube formation, with increased efficacy in the presence of the VEGF during extended treatment.

### 2.6. LA Inhibits VEGF-Induced Microvessel Sprouting in the Rat Aortic Ring Assay

**Figure 8:**
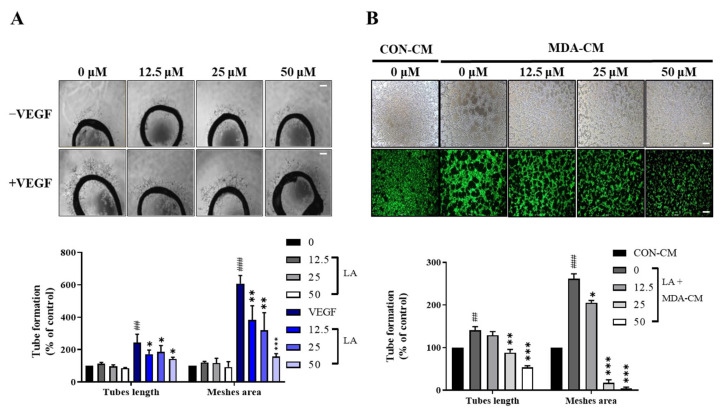
(**A**) LA inhibits VEGF-induced microvessel sprouting ex vivo. Aortas embedded in Matrigel were treated with LA in the presence or absence of 10 ng/mL of the VEGF. After six days of incubation, microvessel growth from the aortic rings was measured using microscopic images. Data are expressed as the mean ± SD of three independent experiments. * *p* < 0.05, ** *p* < 0.01, and *** *p* < 0.001, compared to the vehicle dimethyl sulfoxide (DMSO) control. (**B**) LA inhibits the MDA-MB-231 breast tumor cell-stimulated tube formation of EA.hy926 cells. EA.hy926 cells were pre-incubated with LA for 48 h, followed by an additional incubation for 12 h with LA in media containing 50% MDA-MB-231 conditioned media (MDA-CM) or control-conditioned media (CON-CM). Tube formation was quantified by measuring the average of 4 different images, with representative images shown. Data are expressed as the mean ± SD of three independent experiments. Statistical significance is indicated as * *p* < 0.05, ** *p* < 0.01, and *** *p* < 0.001. LA-only treated groups were compared to the vehicle control (DMSO), while LA + VEGF-treated groups were compared to the VEGF. ## *p* < 0.01, and ### *p* < 0.001, compared to the DMSO control. Scale bar, 100 μm.

To further assess the anti-angiogenic properties of LA, we used an ex vivo rat aortic ring assay (Figure 8A). The VEGF (10 ng/mL) induced complex microvessel sprouting around the aortic rings after six days of incubation. LA significantly decreased the VEGF-induced microvascular length from 172.7% to 15.9%, 14.6%, and 7.6% with 12.5, 25, and 50 μΜ of LA, respectively, in a dose-dependent manner. Additionally, the VEGF-induced microvascular meshes area decreased from 152.5% to 93%, 89.1%, and 82.4%, further confirming that LA effectively inhibits VEGF-induced angiogenesis in an ex vivo model.

### 2.7. LA Inhibits Human Breast Tumor Cell-Induced In Vitro Tube Formation in EA.hy926 Cells

Angiogenesis is crucial for the growth and metastasis of various malignant tumors, including breast, colorectal, and lung cancers [16,17]. To assess the effect of LA on tumor-driven angiogenesis, we used a conditioned medium (MDA-CM) from MDA-MB-231 human breast cancer cells to stimulate endothelial tube formation. MDA-CM mimics breast tumor conditions by enhancing the sprouting capacity of endothelial cells via VEGF signaling [4,19].

As shown in Figure 8B, MDA-CM significantly increased tube formation in EA.hy926 cells compared to control-conditioned media (CON-CM). However, LA treatment inhibited MDA-CM-induced tube formation in a dose-dependent manner. In cells treated with 50 μM of LA, the tube length and mesh area were reduced by up to 86.5% and 256.6%, respectively, compared to the cells treated with MDA-CM. These results suggest that LA suppresses tumor-associated angiogenesis, supporting its potential as an anti-angiogenic agent in cancer therapy.

### 2.8. LA Preferentially Binds to the Active VEGFR2 Conformation

**Figure 9:**
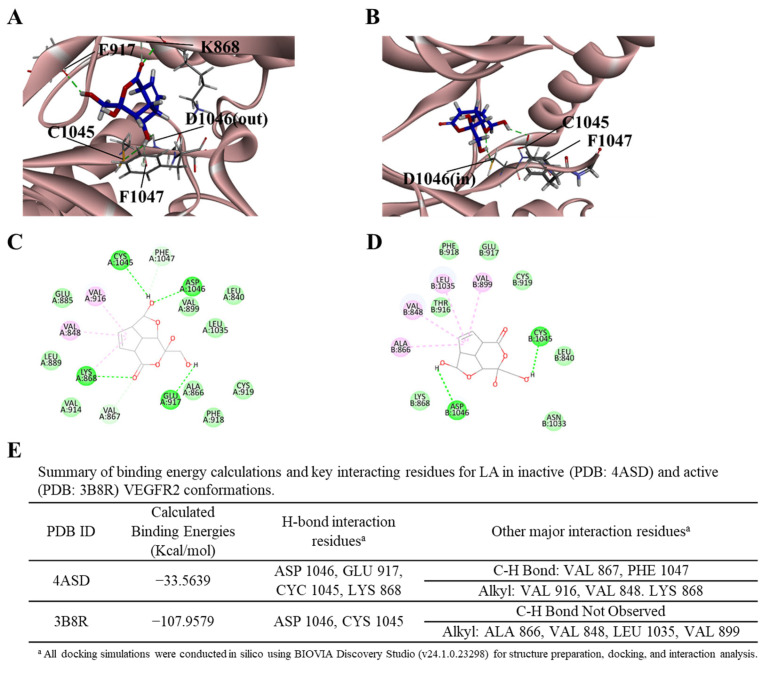
Molecular docking analysis of Lucknolide A (LA) with VEGFR2. (**A**) Docking conformation of LA in the inactive VEGFR2 (DFG-out) conformation (PDB: 4ASD), showing hydrogen bond interactions with D1046 (Asp), C1045 (Cys), K868 (Lys), and E917 (Glu) within the ATP-binding pocket. (**B**) The docking conformation of LA in the active VEGFR2 (DFG-in) conformation (PDB: 3B8R), highlighting interactions with D1046 (Asp) and C1045 (Cys). (**C**) A 2D interaction diagram of LA in the inactive VEGFR2 state, illustrating key molecular interactions. (**D**) A 2D interaction diagram of LA in the active VEGFR2 state, displaying ligand–receptor contacts. (**E**) A summary of the binding energy calculations and key interacting residues for LA in inactive (4ASD) and active (3B8R) VEGFR2 conformations. Hydrogen bond interactions and other key binding residues are listed, showing a significantly stronger binding affinity in the active state (−107.9579 kcal/mol) compared to the inactive state (−33.5639 kcal/mol), suggesting a higher binding preference for the active VEGFR2 conformation.

Finally, to further investigate LA’s mechanism of action on VEGF/VEGFR2 signaling and to explore the molecular basis for the enhanced effects observed with LA and VEGF co-treatment, we performed molecular docking studies to analyze the interactions between LA and VEGFR2 in both its inactive (DFG-out, PDB: 4ASD) and active (DFG-in, PDB: 3B8R) conformations (Figure 9 and Figure S2) [20,21,22,23].

VEGFR2 activation is regulated by the conserved Asp-Phe-Gly (DFG) motif, which transitions between two conformations: DFG-in (active), where Asp1046 enables ATP binding and Phe1047 rotates outward for substrate phosphorylation, and DFG-out (inactive), where a 180° rotation positions Phe1047 inward and Asp1046 outward, preventing ATP binding and stabilizing the kinase in an autoinhibited state [20,21,22,23].

Our results demonstrated that while LA binds to both VEGFR2 states, it exhibits significantly stronger affinity for the active conformation (−107.96 kcal/mol) compared to the inactive state (−33.56 kcal/mol), suggesting its potential role as a VEGFR2 kinase inhibitor. In the inactive VEGFR2 conformation (DFG-out), LA formed hydrogen bonds primarily with Asp1046, Cys1045, Lys868, and Glu917, stabilizing its binding within the ATP-binding pocket (Figure 9A,C). However, Phe1047 remained in an inward-facing position, obstructing ATP binding and preventing kinase activation. In contrast, in the active VEGFR2 conformation (DFG-in), LA exhibited a significantly stronger binding affinity, with Asp1046 and Cys1045 continuing to serve as key interaction sites (Figure 9B,D). Additionally, Phe1047 rotated outward, facilitating ATP coordination and kinase activation. Hydrophobic interactions involving Val848, Leu1035, Val899, and Ala866 further contributed to the increased binding affinity in the active state.

The summary table (Figure 9E) highlights the differences in binding affinity and interaction profiles between the two VEGFR2 conformations. These findings suggest that LA preferentially binds to the active VEGFR2 conformation, potentially disrupting VEGF-induced signaling. This molecular mechanism may explain the enhanced apoptotic response observed upon VEGF co-treatment.

## 3. Discussion

In this study, we investigated the anti-angiogenic effects of Lucknolide A (LA), a natural compound isolated from marine-derived *Streptomyces* sp., and evaluated its potential as a selective inhibitor of VEGF/VEGFR2-mediated angiogenesis. Our results showed that LA effectively inhibits human endothelial cell proliferation, migration, and tube formation—key processes in angiogenesis—with minimal effects on other human cell types, including normal fibroblasts and various tumor cells. LA’s inhibitory effects on endothelial proliferation and tube formation were dose- and time-dependent, with prolonged treatment further enhancing its efficacy. These findings suggest that LA has high specificity for endothelial cells, making it a promising candidate for anti-angiogenic therapy [3,24,25].

Cell cycle analysis revealed that LA induces S-phase cell cycle arrest in endothelial cells, further enhanced by VEGF stimulation. This suggests that LA not only inhibits endothelial proliferation but also sensitizes cells to apoptotic signaling, particularly in the presence of proangiogenic factors such as the VEGF. The increase in apoptotic markers, including p53, Bax, and p21, along with the downregulation of the anti-apoptotic protein Bcl-2, supports the role of LA in promoting apoptotic cell death through intrinsic pathways [4,26]. Furthermore, LA significantly inhibited VEGFR2 phosphorylation and its downstream signaling cascades, including Akt/mTOR/p70S6K, MEK/ERK, Src, FAK, and p38 MAPK. Since VEGFR2 has a central role in VEGF-driven angiogenesis, its suppression by LA provides a molecular basis for its anti-angiogenic effects. The enhanced efficacy observed under VEGF stimulation further reinforces LA’s specificity in targeting VEGFR2 signaling [3,16].

To elucidate LA’s molecular interactions with VEGFR2, we conducted molecular docking studies on both inactive (DFG-out, PDB: 4ASD) and active (DFG-in, PDB: 3B8R) VEGFR2 conformations (Figure 9 and Figure S2)**.** LA binds to both VEGFR2 states but exhibits a significantly stronger affinity for the active conformation (−107.96 kcal/mol) compared to the inactive state (−33.56 kcal/mol), suggesting its potential role as a VEGFR2 kinase inhibitor.

In the inactive VEGFR2 conformation (DFG-out, PDB ID: 4ASD)**,** LA formed stable hydrogen bonds with Asp1046, Cys1045, Lys868, and Glu917, stabilizing its position within the ATP-binding pocket (Figure 9A,C). However, Phe1047 remained in an inward-facing position, obstructing ATP binding and preventing kinase activation. In contrast, in the active VEGFR2 conformation (DFG-in, PDB ID: 3B8R), LA exhibited stronger binding affinity, with Asp1046 and Cys1045 continuing as key interaction sites (Figure 9B,D). Hydrophobic interactions involving Val848, Leu1035, Val899, and Ala866 further contributed to the increased binding affinity in the active state. Additionally, Phe1047 rotated outward, facilitating ATP coordination and kinase activation. The summary table (Figure 9E) highlights the differences in binding affinity and interaction profiles between the two VEGFR2 conformations. These findings suggest that LA preferentially binds the active VEGFR2 conformation, potentially disrupting VEGF-induced signaling. This molecular mechanism explains LA’s enhanced effects under VEGF stimulation.

To further validate the specificity of LA’s action, we compared its docking properties with Lucknolide B (LB), a structural analog of LA co-isolated by Yadav et al. [15]. LB shares the same tricyclic structural scaffold as LA, except for a methoxyl group at C8 instead of a hydroxyl group (Supplementary Figure S5). Docking analysis revealed that LA exhibited a significantly stronger binding affinity for the active VEGFR2 (−107.96 kcal/mol) compared to LB (−73.67 kcal/mol). Furthermore, while LA successfully docked to both the inactive (DFG-out) and active (DFG-in) VEGFR2 conformations, LB failed to bind to the inactive VEGFR2 conformation (Figure 9 and Supplementary Figure S5). In the inactive VEGFR2 state (DFG-out, PDB ID: 4ASD), LA formed stable hydrogen bonds with key ATP-binding residues, including Asp1046, Cys1045, Lys868, and Glu917, within the kinase domain (Supplementary Figure S5A,B,D,F,G). In contrast, LB did not achieve docking with the inactive VEGFR2 state, reinforcing its lower affinity. Structural comparisons suggest that LB’s failure to dock to the inactive state may be due to steric repulsion at Asp1046, which impairs stable binding. These findings confirm the specificity of LA’s inhibitory activity, as LB—despite its high structural similarity—exhibits significantly weaker VEGFR2 binding affinity. This suggests that LA’s effects arise from its unique binding properties rather than the general characteristics of the tricyclic scaffold.

Additionally, we also examined the docking profiles of three well-known VEGFR2 inhibitors—sunitinib [27,28], sorafenib [28], and tivozanib [29]—to contextualize LA’s binding characteristics (Supplementary Figure S6). Sunitinib, a type I inhibitor, exhibited a high affinity for the active VEGFR2 conformation, whereas sorafenib and tivozanib, both type II inhibitors, showed comparable binding affinities between inactive and active states. Notably, LA demonstrated a strong binding affinity for the active VEGFR2 conformation, suggesting a type I inhibitor-like interaction. Furthermore, LA exhibited a significant increase in binding affinity between the inactive (−33.56 kcal/mol) and active (−107.96 kcal/mol) states, surpassing the affinity shift observed for sunitinib. Additionally, LA retained hydrogen bond interactions with key residues in the DFG motif, even in the inactive state. These findings suggest that LA preferentially binds the active (DFG-in) VEGFR2 conformation, behaving similarly to type I inhibitors. However, the substantial shift in binding energy and its partial engagement with the DFG motif in the inactive state also suggest a possible alternative inhibitory mechanism distinct from conventional type I VEGFR2 inhibitors.

Collectively, our docking studies indicate LA’s specificity as a VEGFR2 inhibitor, with significantly stronger binding affinity compared to LB despite their structural similarity. LA behaves similarly to type I inhibitors by preferentially binding the active VEGFR2 conformation, yet its substantial binding energy shift and partial engagement with the DFG motif suggest an alternative inhibitory mechanism. Further studies are required to clarify this mechanism and its biological implications.

To assess LA’s specificity in VEGF/VEGFR2 signaling suppression, we also examined its effects under FGF and PDGF stimulation in addition to the VEGF. LA strongly inhibited VEGF-induced endothelial proliferation, whereas it had no significant effect on FGF- or PDGF-stimulated cell growth, suggesting that LA selectively targets VEGF-driven endothelial proliferation conditions (Supplementary Figure S3). However, in the tube formation assay, we found that while LA strongly suppressed VEGF-induced tube formation, it also moderately reduced tube formation under FGF- and PDGF-stimulated conditions (Supplementary Figure S4). This discrepancy may be due to the complexity of tube formation, which involves migration, adhesion, and cytoskeletal remodeling beyond proliferation. Additionally, crosstalk between VEGF, FGF, and PDGF signaling pathways, structural similarities in their receptor tyrosine kinases, and differences in receptor expression levels could contribute to differential effects. Further investigation is needed to fully elucidate LA’s influence on VEGFR, FGFR, and PDGFR signaling pathways. Nonetheless, our results indicate that LA selectively inhibits VEGF/VEGFR2-mediated endothelial proliferation and tube formation.

Moreover, LA inhibited VEGF-induced microvessel sprouting in an ex vivo rat aortic ring assay and suppressed tube formation induced by human breast tumor cells. The inhibitory effect of LA on MDA-MB-231-stimulated tube formation further enhances LA’s potential as a therapeutic agent in tumor treatment, particularly in cancers that are highly dependent on VEGF signaling-induced angiogenesis, such as breast cancer [4,19].

The specificity of LA for endothelial cells, along with its selective inhibition of VEGF/VEGFR2 signaling, distinguishes it from broad-spectrum anti-angiogenic agents that often cause off-target effects and toxicity [5,6,30]. While existing agents, such as bevacizumab and sunitinib, are effective, they are often associated with severe side effects and resistance development, limiting their long-term clinical use. LA’s selective targeting of VEGF/VEGFR2 signaling may provide a more targeted and potentially safer alternative. However, further studies are required to elucidate LA’s in vivo efficacy, pharmacokinetics, and toxicity. Additionally, structure–activity relationship (SAR) studies may help optimize LA’s anti-angiogenic properties for clinical development.

In conclusion, LA is a promising marine-derived compound with potent anti-angiogenic activity. It selectively inhibits endothelial cell proliferation, migration, and tube formation by suppressing VEGF/VEGFR2 signaling while minimally affecting other pathways. Additionally, LA suppresses tumor-associated angiogenesis, suggesting its potential as a therapeutic agent for VEGF-dependent cancers. Further research on LA’s molecular interactions, in vivo efficacy, and therapeutic potential may contribute to the development of selective anti-angiogenic agents for cancer and other angiogenesis-related diseases.

## 4. Materials and Methods

### 4.1. Chemicals

An EA 3-(4,5-Dimethyl-2-yl)-2,5-diphenyltetrazolium bromide (MTT) reagent was obtained from Sigma-Aldrich Chemical Co. (St. Louis, MO, USA). VEGF-A was purchased from GeneScript in Piscataway, NJ, USA. Antibodies used for Western blot analysis were purchased from Santa Cruz Biotechnology, Inc. (Santa Cruz, CA, USA) and Cell Signaling Technology (Danvers, MA, USA). All other chemicals and reagents were of analytical and biological grade and commercially available.

### 4.2. Extraction and Isolation of LA

The extraction and isolation of Lucknolide A (LA) from *Streptomyces* sp. 151KO-065 was performed as described in our previous study [14], with minor modifications. Briefly, *Streptomyces* sp. 151KO-065 was cultured on Bennett’s (BN) agar plates and was then scaled up in a BN medium in progressively larger flasks and a 100 L fermenter. After 7 days of incubation, the culture broth was separated by centrifugation. The supernatant was extracted with EtOAc, concentrated, and subjected to flash column chromatography with a MeOH/H_2_O gradient. Final purification of the target compound was achieved using a C18 reversed-phase HPLC, yielding 125 mg of LA.

### 4.3. Cell Culture and Cell Proliferation Assay

EA.hy926 human endothelial cells were acquired from the American Type Culture Collection (Manassas, VA, USA). Dulbecco’s Modified Eagle’s medium (DMEM) and fetal bovine serum (FBS) were purchased from WELGENE (Gyeongsan-si, Republic of Korea), and penicillin/streptomycin was purchased from Gibco BRL (Life Technology, New York, NY, USA). EA.hy926 cells were grown in DMEM media containing 10% FBS and 100 U/mL of penicillin/100 μg/mL streptomycin and incubated at 37 °C in a humidified atmosphere 5% of CO_2_. Human umbilical endothelial cells (HUVECs) and M200 media with Large Vessel Endothelial Supplement (LVES) were purchased from Thermo Fisher Scientific, Waltham, MA, USA. HUVEC cells were grown in M200 media containing 2% LVES and incubated at 37 °C in a humidified atmosphere of 5% CO_2_. The cells were cultured every 2–3 days and seeded in 96-well plates to determine the anti-proliferative effect of LA. Cells (8 × 10^3^ cells/well) were seeded and incubated overnight. For the experimental procedure, the cells were treated with varying concentrations of LA (0–50 μM) in the presence or absence of VEGF-A, FGF-2, and PDGF-bb (50 ng/mL at final concentration), followed by incubation for different time intervals at 37 °C under a humidified atmosphere of 5% CO_2_. After each round of incubation, 100 μL of MTT solution (0.5 mg/mL) was added to each well and incubated for another 4 h at 37 °C. Subsequently, the culture medium was removed, and 100 μL of dimethyl sulfoxide (DMSO) was added to each well to dissolve the formazan crystals. The plates were then incubated on a shaker for 30 min at room temperature (RT). After thorough mixing, the absorbance was measured at 595 nm using a microplate reader (FilterMax F5, Molecular Devices, Silicon Valley, CA, USA).

### 4.4. Cell Cycle Arrest and Apoptosis Analysis

Cells were incubated with or without LA at concentrations of 0–50 μM for 3 days in the presence or absence of VEGF-A in a CO_2_ incubator. Following incubation, the cells were harvested using phosphate-buffered saline (PBS) and centrifuged at 1000 rpm for 3 min. After discarding the supernatant, the cell pellet was fixed with ice-cold 70% ethanol (*v*/*v*) over 3 h at −20 °C. After fixation, cells were washed twice with PBS. Subsequently, 100 μL of PBS and an equal volume of propidium iodide (PI) solution containing 50 μg/mL of RNase A were added to each tube. The cells were then incubated in the dark for 30 min at RT. At the end of the incubation period, the stained cells were resuspended and loaded onto a flow cytometer (FACS verse, Becton Dickinson, Heidelberg, Germany).

### 4.5. Observation of Apoptotic Cells of EA.hy926 Cells

Apoptotic cells were visualized using phase contrast and fluorescence microscopy, followed by staining with Hoechst 33,258, as outlined by Naito [31]. Cells were cultured in 24-well plates at 8 × 10^4^ cells. After overnight incubation, the cells were treated with varying concentrations of LA and further incubated for 72 h. At the end of the incubation period, the cells were washed twice with 1× PBS and fixed with 3.7% formaldehyde in PBS for 20 min at RT. The fixed cells were washed three times with PBS and then stained with 0.12 mg/mL of Hoechst 33,258 for 20 min at RT. Subsequently, the stained cells were washed with PBS. A fluorescence microscope (Motic AE31, MHG-100B, Jed Pella Co., Redding, CA, USA) was used to observe and photograph the stained cells.

### 4.6. Wound Healing Assay

The cells (2 × 10^4^ cells/well) were seeded in 48-well plates and incubated overnight. EA.hy926 cells were treated with various concentrations (0–50 μM) of LA in EA.hy926 cells with growth factor reduced serum-free media (Medium 200) and pre-incubated for 48 h. After that, the cells were scratched with a 200 μL tip and washed with PBS 3 times, followed by retreatment with different concentrations of LA in the absence or presence of 50 ng/mL VEGFA. Images were captured at 0–24 h of wound healing. The length of the wound in each group was measured using the ImageJ software (v1.54f). The scratched area was calculated 18 h after the wounding area at 0 h. The results were normalized for comparison with those of the vehicle control group.

### 4.7. Tube Formation Assay

The day before performing the tube formation assay, the growth factor reduced basement membrane Matrigel (Corning, Cornyn, NY, USA) matrix was incubated at 4 °C overnight. On the day of the experiment, the Matrigel was transferred to each well of a μ-slide chamber, which was then incubated at 37 °C for 1 h. The prepared cells were seeded onto the solidified Matrigel. Cells were seeded in 6-well plates at a density of 2 × 10^5^ cells/mL and incubated overnight. LA was added at various concentrations in a serum-free medium and incubated for 48 h. At the end of the incubation period, the cells were detached using a trypsin–EDTA buffer, collected by centrifugation, and resuspended. The resuspended cells were seeded into a μ-slide chamber at a density of 1 × 10^4^ cells/well. They were further treated with LA in the absence or presence of VEGF-A, FGF-2, and PDGF-bb (50 ng/mL) for 12 h and stained with a Calcein–AM solution (final 2 μg/mL) during 30 min incubation. Tube-like structures formed by the cells were analyzed using the AngioTool software (https://angiotool.software.informer.com/0.6/).

To prepare a conditioned medium of MDA-MB-231 breast tumor cells (MDA-CM), MDA-MB-231 cells were cultured in DMEM containing 10% FBS and 100 U/mL of penicillin/100 μg/mL streptomycin until they reached 80% confluence. The cells were then washed with PBS and incubated for an additional 2 days in a growth medium supplemented with 2 mM L-glutamine. For the control conditioned medium (CON-CM), DMEM was incubated under cell-free conditions for three days. The MDA-CM and CON-CM were filtered through a 0.2 μm filter (Corning, Cornyn, NY, USA), aliquoted, and stored at −80 °C until use.

### 4.8. Rat Aorta Ring Assay

The 48-well tissue culture plates were covered with 150 µL of Matrigel and allowed to solidify for 30 to 45 min at 3 7 °C in a 5% CO_2_ incubator. The isolation of rat aortas from Sprague–Dawley (SD) rats followed the procedures described in previous publications [32,33]. Thoracic aortas were excised from 7-week-old male SD rats (Orient Bio, Seongnam, Republic of Korea), and the surrounding fibroadipose tissue was carefully removed. The aortas then were sectioned into 1–1.5 mm-long cross-sections, rinsed multiple times with PBS, placed on the solidified Matrigel in the wells, and covered with an additional 100 µL of Matrigel. The aortic rings were cultured in serum-free M199 medium for 24 h. Following incubation, the medium was replaced with a complete M199 medium (1.5 mL) containing the indicated concentrations of LA. The medium was refreshed every two days. After six days of incubation, the rings were fixed with 4% paraformaldehyde (PFA), and microvessel growth was measured using photographs taken with a CKX41 Olympus microscope with a ×40 objective. The ex vivo experiments involving animal vascular tissues were outsourced to EBO, an external experimental service provider. EBO conducted all procedures in compliance with ethical guidelines and under the approval of their Institutional Animal Care and Use Committee (IACUC). Details of the ethics approval are provided below.

### 4.9. Western Blotting Analysis

Western blotting was performed according to the standard procedures. Briefly, EA.hy926 cells were cultured at a density of 2 × 10^5^ cells/well in 6-well plates and incubated overnight to allow attachment to the bottom of the plate. Various concentrations of LA in serum-free medium were added to each well and pre-incubated for 3 days in the absence or presence of 50 ng/mL of VEGF-A. After treatment, the cells were washed twice with PBS and lysed in a radioimmunoprecipitation assay (RIPA) buffer at 4 °C. The total protein was extracted, separated using 10% sodium dodecyl sulfate–polyacrylamide gel electrophoresis and 5% stacking gels, and transferred onto a PVDF membrane (Immobilon-P, Millipore, Burlington, MA, USA). The membrane was blocked for 1h at RT using phosphate-buffered saline and tween 20 (PBS-T) containing 5% skim milk and 1% bovine serum albumin (BSA). After washing with PBS-T, the membrane was incubated overnight with the appropriate primary antibodies and alkaline phosphatase-conjugated or horseradish peroxidase (HRP)-conjugated secondary antibodies for 30 min. After incubation, protein bands were detected using the NBT/BCIP solution or a chemilumines cent ECL assay kit (Amersham Pharmacia, Buckinghamshire, UK). Immunoblotted bands were visualized using a LAS-3000 system and quantified using the MultiGauge V 3.0 software (Fujifilm Life Science, Tokyo, Japan).

### 4.10. Molecular Docking

The three-dimensional (3D) structures of VEGFR-2 used in docking studies were obtained from the Protein Data Bank (PDB). Molecular docking was performed using Discovery Studio Client (v24.1.0.23298), employing three known tyrosine kinase inhibitors (sorafenib, tivozanib, and sunitinib) as reference compounds, alongside two candidate compounds (Lucknolide A and Lucknolide B). The docking procedure aimed to calculate binding energies and determine the most favorable binding poses for further structural and energetic analyses. Docking results were evaluated based on binding energy scores, and the most optimal poses were selected based on 2D interaction diagrams and ligand conformations. The receptor–ligand interactions were assessed to identify key hydrogen bonding, hydrophobic interactions, and other stabilizing forces within the ATP-binding pocket. The binding sites of the co-crystallized proteins were determined through the self-docking of each reference inhibitor.

### 4.11. Protein and Ligand Prepare

Prior to docking, the ligands were prepared by retrieving their 3D structures in the SDF format from PubChem. The ligand structures underwent energy minimization using the full minimization method (CHARMM36 [34,35]) in Discovery Studio Client (v24.1.0.23298) to obtain optimized conformations suitable for docking. These compounds are referred to by their designated numbers in the subsequent sections for clarity and consistency. Protein preparation was performed using Discovery Studio Visualizer. This involved the removal of water molecules, heteroatoms, and co-crystallized ligands from the crystal structures, followed by PDB format conversion for docking simulations. To validate the docking accuracy, self-docking (re-docking) was performed, and the root-mean-square deviation (RMSD) between the re-docked and crystallized ligand conformations was calculated, a widely accepted method for docking validation [36,37]. The docking protocol was considered valid if the RMSD was ≤1.0 Å, ensuring reliable ligand positioning within the binding pocket. The docking results were further validated by comparing the docking poses of known VEGFR2 inhibitors (sorafenib, tivozanib, and sunitinib) with their co-crystal structures in PDB. The re-docked conformations closely aligned with the experimental structures, further supporting the reliability of the docking methodology.

### 4.12. Chemical Identification of Ligands (PubChem IDs)

Sorafenib (Compound CID: 216239), tivozanib (Compound CID: 9911830), sunitinib (Compound CID:5329102), N-cyclopropyl-6-[(6,7-dimethoxyquinolin-4-yl)oxy]naphthalene-1-carboxamide (Compound CID:11441375). The structures of Lucknolide A (LA) and Lucknolide B (LB) were adopted from a previous study [15].

### 4.13. Statistical Analysis

Statistical analysis of the data was performed using the InStat statistical program (3.0, GraphPad Software, Inc., San Diego, CA, USA). To examine statistical variance, we used a one-way analysis of variance (ANOVA) with Tukey’s multiple comparison test.

## 5. Conclusions

Our study demonstrates that Lucknolide A (LA), a marine *Streptomyces*-derived compound, exhibits potent anti-angiogenic activity by selectively targeting endothelial cells. LA effectively inhibited endothelial cell proliferation, induced S-phase cell cycle arrest, and promoted apoptosis, particularly under VEGF stimulation. Additionally, LA suppressed key angiogenic processes, including cell migration, tube formation, and microvessel sprouting, both in vitro and ex vivo. These anti-angiogenic effects were associated with the inhibition of VEGFR2 phosphorylation and its downstream signaling pathways, including Akt/mTOR/p70S6K, MEK/ERK, Src, FAK, and p38 MAPK. Molecular docking studies further revealed that LA preferentially binds to the active VEGFR2 conformation (DFG-in) with significantly higher affinity (−107.96 kcal/mol) compared to the inactive state (−33.56 kcal/mol), suggesting its potential as a selective VEGFR2 inhibitor. The selective inhibition of endothelial cells, coupled with its high VEGFR2 binding specificity, suggests that LA is a promising candidate for anti-angiogenic therapy. Given its effectiveness in suppressing VEGF-driven angiogenesis and tumor-associated tube formation, LA has potential as a therapeutic agent for angiogenesis-related diseases, including cancer. Further investigation into its in vivo efficacy, pharmacokinetics, and toxicity in animal models is essential to assess its therapeutic potential and clinical applicability.

## Data Availability

The data that support the findings of this study are available from the corresponding author (S.H.J. and S.J.P.) upon reasonable request.

## Supplementary Materials

The following supporting information can be downloaded at: https://www.mdpi.com/article/10.3390/molecules30050987/s1, Figure S1. LA inhibits VEGF-induced tube formation of HUVEC cells. HUVEC cells were pre-treated with at 0, 12.5, 25, and 50 μM LA for 48 h and then further incubated with LA for 12 h in the presence or absence of 50 ng/ml VEGF. Tube formation was quantified by measuring the average of 3 different images, and representative images are shown. Data are expressed as the mean ± SD of three independent experiments. Statistical significance is indicated as * *p* < 0.05, ** *p* < 0.01, and *** *p* < 0.001. LA-only treated groups were compared to the vehicle control (DMSO), while LA + VEGF-treated groups were compared to the VEGF. # *p* < 0.05, ## *p* < 0.01, and ### *p* < 0.001, compared to DMSO control. Scale bar, 100 μm. Figure S2. Structural comparison of the inactive (DFG-out) and active (DFG-in) VEGFR2 conformations. (A) Inactive VEGFR2 conformation (DFG-out, PDB: 4ASD): Asp1046 is oriented outward, while Phe1047 is positioned inward within the kinase domain, blocking the ATP-binding site and preventing kinase activation. (B) Active VEGFR2 conformation (DFG-in, PDB: 3B8R): Asp1046 is oriented inward, facilitating ATP coordination, while Phe1047 rotates outward, enabling substrate phosphorylation. The VEGFR2 protein structure is shown in light red, with the DFG motif highlighted in distinct colors: carbon atoms in dark gray, oxygen in red, and hydrogen in light gray. "α-c" refers to the αC-helix. Figure S3. LA decreases the proliferation of EA.hy926 cells in a dose- and time-dependent manner. EA.hy926 cells were treated with 0–50 µM LA in the presence or absence of 50 ng/ml VEGF, FGF2, or PDGF-bb for different time intervals (24, 72 h, and 120 h). CC50 (μM) is expressed as the mean ± standard deviation (SD) of three independent experiments. Statistical significance is indicated as * *p* < 0.05, ** *p* < 0.01, and *** *p* < 0.001. LA-only treated groups were compared to the vehicle control (DMSO), while LA + Growth Factor-treated groups were compared to the Growth Factor. Figure S4. LA inhibits growth factor (G,F)-induced tube formation of EA.hy926 cells. EA.hy926 cells were pre-treated at specific concentrations of LA for 48 h and then further incubated with LA for 12 h in the presence or absence of 50 ng/ml VEGF, FGF2, or PDGF-bb. Tube formation was quantified by measuring the average of 3 different images, and representative images are shown. Data are expressed as the mean ± SD of three independent experiments. Statistical significance is indicated as * *p* < 0.05, ** *p* < 0.01, and *** *p* < 0.001. LA-only treated groups were compared to the vehicle control (DMSO), while LA + Growth factor-treated groups were compared to the Growth factor. # *p* < 0.05, ## *p* < 0.01, and ### *p* < 0.001, compared to DMSO control. Scale bar, 100 μm. Figure S5. Molecular docking analysis of Lucknolide A (LA) and Lucknolide B (LB) with VEGFR2. (A–C) Docking conformations of LA and LB in VEGFR2, while (D–F) illustrate their respective 2D ligand-receptor interactions. (A) Docking conformation of LA in the inactive VEGFR2 (DFG-out) conformation (PDB: 4ASD). (B) Docking conformation of LA in the active VEGFR2 (DFG-in) conformation (PDB: 3B8R). (C) Docking conformation of LB in the active VEGFR2 (DFG-in) conformation (PDB: 3B8R). (D) 2D interaction diagram of LA in the inactive VEGFR2 state, showing key ligand-receptor interactions. (E) 2D interaction diagram of LA in the active VEGFR2 state, highlighting hydrogen bonding and hydrophobic interactions. (F) 2D interaction diagram of LB in the active VEGFR2 state, displaying ligand-receptor interactions. Notably, docking analysis revealed that LB did not bind to the inactive VEGFR2 conformation (PDB: 4ASD). The red-colored interactions in the 2D diagram indicate atomic repulsion forces, suggesting that LB experiences repulsive interactions with the DFG motif, which may explain the lack of a significant increase in binding energy compared to LA (G) Molecular interaction of human VEGFR2 active site (4ASD, 3B8R) with LA and LB. Figure S6. (A) Molecular interaction of the human inactive site VEGFR2 (4ASD) with LA and reported inhibitors. (B) Molecular interaction of the human active site VEGFR2 (3B8R) with LA and reported inhibitors.
