# Supplementary material for: Anti-Angiogenic Potential of Marine Streptomyces-Derived Lucknolide A on VEGF/VEGFR2 Signaling in Human Endothelial Cells
Source: Molecules. 2025 Feb 20;30(5):987. doi: 10.3390/molecules30050987 (PMC11901821; doi:10.3390/molecules30050987)
Supplement: Supplementary file 1 [file molecules-30-00987-s001.zip › molecules-3457298-supplementary.pdf]

Supplementary Figure S1.

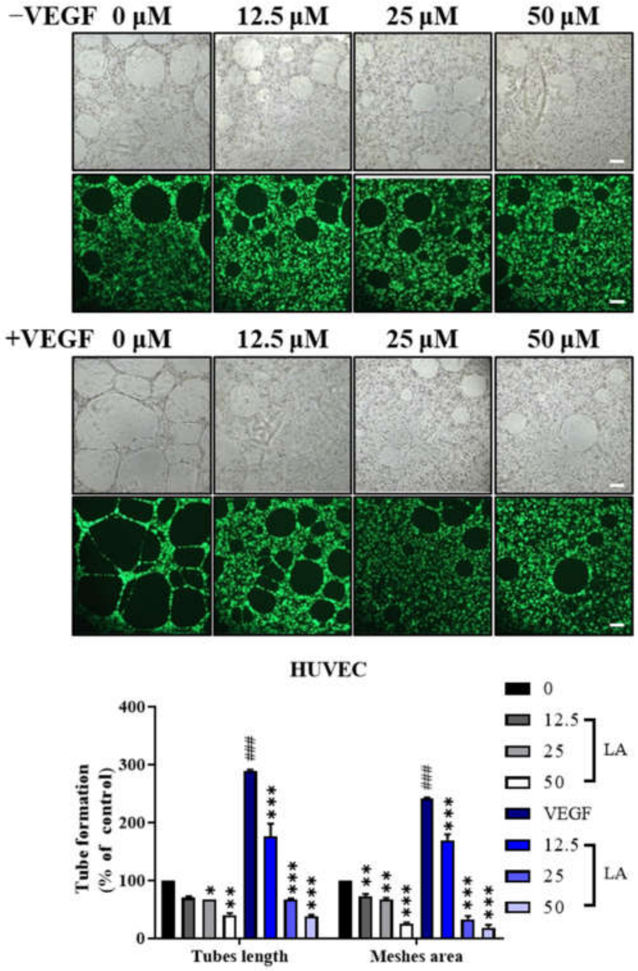

Supplementary Figure S1. LA inhibits VEGF-induced tube formation of HUVEC cells. HUVEC cells were pre-treated with at 0, 12.5, 25, and 50 µM LA for 48 h and then further incubated with LA for 12 h in the presence or absence of 50 ng/ml VEGF. Tube formation was quantified by measuring the average of 3 different images, and representative images are shown. Data are expressed as the mean ± SD of three independent experiments. Statistical significance is indicated as $*p < 0.05$, $**p < 0.01$, and $***p < 0.001$. LA-only treated groups were compared to the vehicle control (DMSO), while LA + VEGF-treated groups were compared to the VEGF. #p < 0.05, ##p < 0.01, and ###p < 0.001, compared to DMSO control. Scale bar, 100 µm.

Supplementary Figure S2.

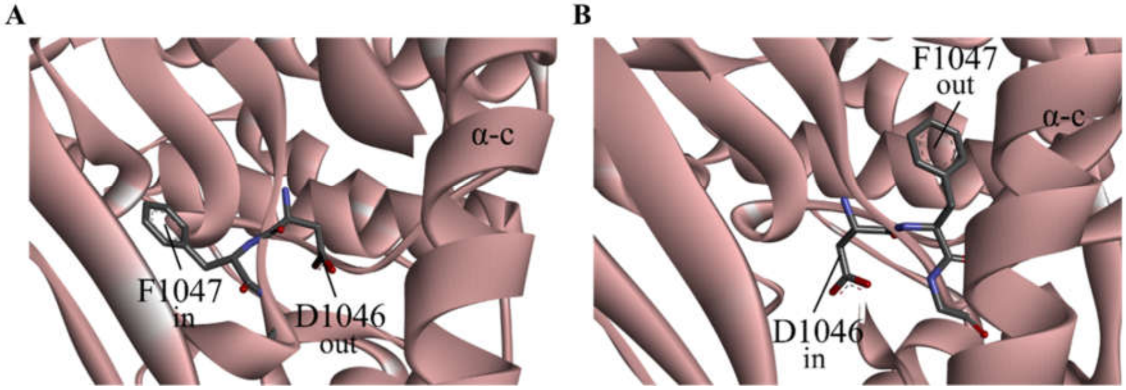

Supplementary Figure S2. Structural comparison of the inactive (DFG-out) and active (DFG-in) VEGFR2 conformations. (A) Inactive VEGFR2 conformation (DFG-out, PDB: 4ASD): Asp1046 is oriented outward, while Phe1047 is positioned inward within the kinase domain, blocking the ATP-binding site and preventing kinase activation. (B) Active VEGFR2 conformation (DFG-in, PDB: 3B8R): Asp1046 is oriented inward, facilitating ATP coordination, while Phe1047 rotates outward, enabling substrate phosphorylation. The VEGFR2 protein structure is shown in light red, with the DFG motif highlighted in distinct colors: carbon atoms in dark gray, oxygen in red, and hydrogen in light gray. "α-c" refers to the αC-helix.

Supplementary Figure S3.

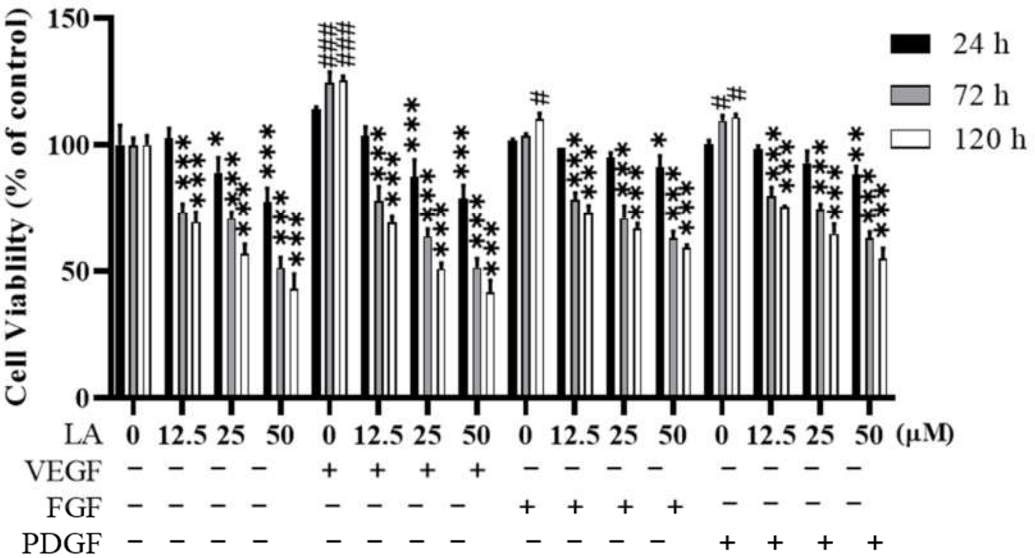

CC_{50} (μM)	−	+ VEGF	+ FGF	+ PDGF
24 h	>50	>50	>50	>50
72 h	40.51±2.3	20.97±3.1	>50	>50
120 h	27.71±1.2	19.48±1.3	36.14±2.0	29.15±1.7

Supplementary Figure S3. LA decreases the proliferation of EA.hy926 cells in a dose- and time-dependent manner. EA.hy926 cells were treated with 0–50 μM LA in the presence or absence of 50 ng/ml VEGF, FGF2, or PDGF-bb for different time intervals (24, 72h, and 120 h). CC_{50} (μM) is expressed as the mean ± standard deviation (SD) of three independent experiments. Statistical significance is indicated as *p < 0.05, **p < 0.01, and ***p < 0.001. LA-only treated groups were compared to the vehicle control (DMSO), while LA + Growth Factor-treated groups were compared to the Growth Factor.

Supplementary Figure S4.

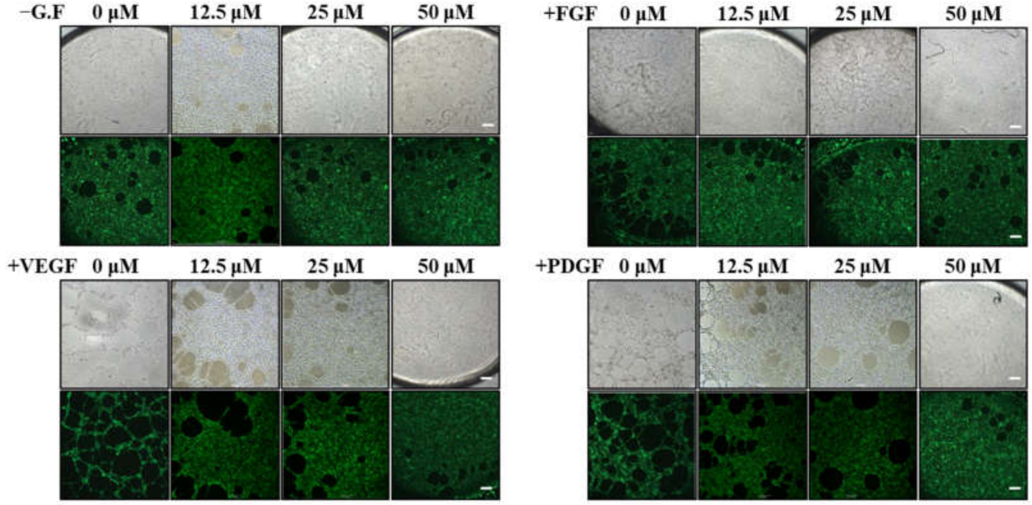

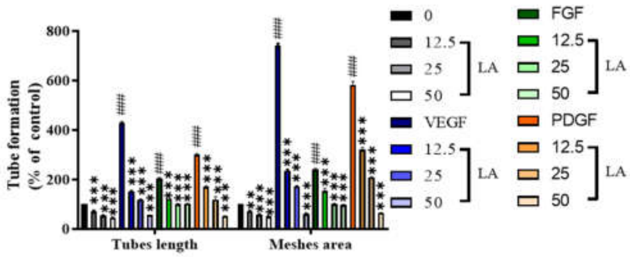

IC$_{50}$ (μM)	− G.F	+ VEGF	+ FGF	+ PDGF
Length	39.43±2.0	7.816±0.1	29.22±0.1	15.00±3.0
Mesh	33.98±2.6	6.191±0.1	23.24±0.7	12.94±0.9

Supplementary Figure S4. LA inhibits growth factor (G.F)-induced tube formation of EA.hy926 cells. EA.hy926 cells were pre-treated at specific concentrations of LA for 48 h and then further incubated with LA for 12 h in the presence or absence of 50 ng/ml VEGF, FGF2, or PDGF-bb. Tube formation was quantified by measuring the average of 3 different images, and representative images are shown. Data are expressed as the mean ± SD of three independent experiments. Statistical significance is indicated as *p < 0.05, **p < 0.01, and ***p < 0.001. LA-only treated groups were compared to the vehicle control (DMSO), while LA + Growth factor-treated groups were compared to the Growth factor. #p < 0.05, ##p < 0.01, and ###p < 0.001, compared to DMSO control. Scale bar, 100 μm.

Supplementary Figure S5.

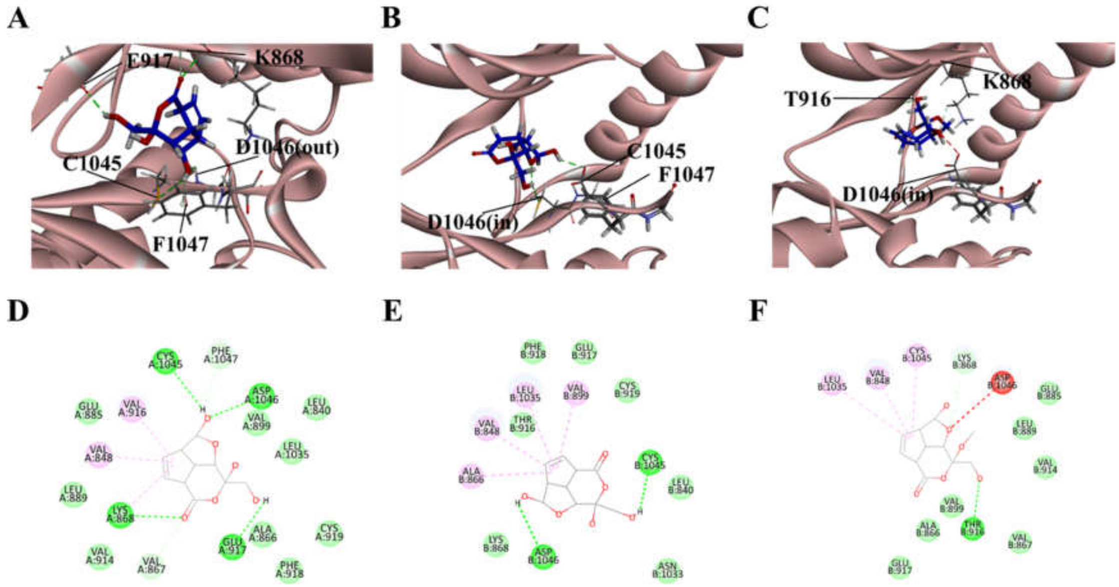

G

Molecular interaction of human VEGFR2 with LA and LB (inactive 4ASD and active 3B8R).

compound	Structure	Calculated Binding Energies (Kcal/mol)	H-bond interaction residues[a]	Other major interaction residues[a]
		**Protein ID : 4ASD**		
LA (MW:228.2g/mol)	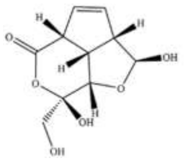	−33.5639	ASP 1046, CYC 1045, LYS 868, GLU 917	C-H Bond: PHE 1047, VAL 867<br>Alkyl: LYS 868, VAL 848, VAL 916
LB (MW:242.2g/mol)	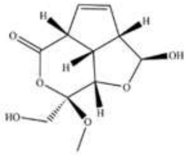	Docking Not Achieved		
		**Protein ID : 3B8R**		
LA (MW:228.2g/mol)	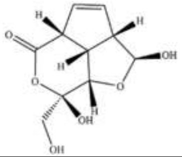	−107.9579	ASP 1046, CYS 1045	Alkyl: ALA 866, VAL 848, LEU 1035, VAL 899
LB (MW:242.2g/mol)	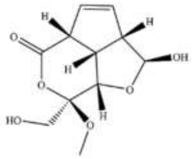	−73.668	THR 916	C-H Bond: LYS 868<br>Alkyl: LEU 1035, VAL 848, CYS 1045<br>Unfavorble Bond: ASP 1046

[a] All docking simulations were conducted in silico using BIOVIA Discovery Studio (v24.1.0.23298) for structure preparation, docking, and interaction analysis.

Supplementary Figure S5. Molecular docking analysis of Lucknolide A (LA) and Lucknolide B (LB) with VEGFR2. (A-C) Docking conformations of LA and LB in VEGFR2, while (D-F) illustrate their respective 2D ligand-receptor interactions. (A) Docking conformation of LA in the inactive VEGFR2 (DFG-out) conformation (PDB: 4ASD). (B) Docking conformation of LA in the active VEGFR2 (DFG-in) conformation (PDB: 3B8R). (C) Docking conformation of LB in the active VEGFR2 (DFG-in) conformation (PDB: 3B8R). (D) 2D interaction diagram of LA in the inactive VEGFR2 state, showing key ligand-receptor interactions. (E) 2D interaction diagram of LA in the active VEGFR2 state, highlighting hydrogen bonding and hydrophobic interactions. (F) 2D interaction diagram of LB in the active VEGFR2 state, displaying ligand-receptor interactions. Notably, docking analysis revealed that LB did not bind to the inactive VEGFR2 conformation (PDB: 4ASD). The red-colored interactions in the 2D diagram indicate atomic repulsion forces, suggesting that LB experiences repulsive interactions with the DFG motif, which may explain the lack of a significant increase in binding energy compared to LA (G) Molecular interaction of human VEGFR2 active site (4ASD, 3B8R) with LA and LB.

Supplementary Figure S6.

A

Molecular interaction of the human inactive site VEGFR2 (4ASD) with LA and reported inhibitors.

Compound	Calculated Binding Energies (Kcal/mol)	H-bond interaction residues[a]	Other major interaction residues[a]
LA (MW:228.2g/mol)	−33.5639	ASP 1046, GLU 917, CYC 1045, LYS 868	C-H Bond: VAL 867, PHE 1047
			Alkyl: VAL 916, VAL 848
Sunitinib[b] (MW:398.47g/mol)	−34.4275	ASP 1046, GLU 917	C-H Bond: GLU 885
			Alkyl: LEU 1035, ALA 866, CYS 919, VAL 916, VAL 914, VAL 848, PHE 1047, LEU 840, LEU 889
Sorafenib[c,d] (MW: 464.825g/mol)	−76.1417	ASP 1046, GLU 885	C-H Bond: HIS 1026, CYS 1045, GLU 917, CYS 919
			Alkyl: HIS 1026, LEU 1019, LEU 1035, ALA 866, LEU 840, PHE 918, CYS 919, VAL 848, VAL 899, VAL 916, LEU 889
Tivozanib[c] (MW:454.87g/mol)	−64.9716	ASP 1046, GLU 885, CYC 919	C-H Bond: PHE 918, GLU 917, CYC 1045, LEU 840, CYS 919
			Alkyl: LEU 1035, ALA 866, VAL 914, LYS 868, VAL 916, LEU 889, LEU 1019, HIS 1026, PHE 1047, LEU 840, PHE 918

[a] All docking simulations were conducted in silico using BIOVIA Discovery Studio (v24.1.0.23298) for structure preparation, docking, and interaction analysis.

[b] Reported type-I VEGFR2 inhibitor. [c] Reported type-II VEGFR2 inhibitor

[d] Root mean square deviation (RMSD) was calculated by comparing the re-docked and crystallized conformations of sorafenib, a value of 0.85Å.

B

Molecular interaction of the human active site VEGFR2 (3B8R) with LA and as reported inhibitors.

Compound	Calculated Binding Energies (Kcal/mol)	H-bond interaction residues[a]	Other major interaction residues[a]
LA (MW:228.2g/mol)	−107.9579	ASP 1046, CYS 1045	Alkyl: ALA 866, VAL 848, LEU 1035, VAL 899
Sunitinib[b] (MW:398.47g/mol)	−69.6764	ASP1046	C-H Bond: CYS 1045, GLU 885, VAL 899, PHE 1047
			Alkyl: LEU 840, LEU 1035, VAL 848, ALA 866, LYS 868, LEU 1049, LEU 889
Sorafenib[c] (MW: 464.825g/mol)	−57.7347	PHE 1047, LYS 868	C-H Bond: ASP 1046, GLU 885
			Alkyl: LEU 840, VAL 848, ALA 866, CYS 1045, VAL 899, LEU 889, ILE 888, LEU 1049
Tivozanib[c] (MW:454.87g/mol)	−62.8056	LYS 868	C-H Bond: LYS 920, LEU 840
			Alkyl: LEU 1035, CYS 919, ALA 866, VAL 848, LEU 889, LEU 1049, PHE 1047, ILE 888

[a] All docking simulations were conducted in silico using BIOVIA Discovery Studio (v24.1.0.23298) for structure preparation, docking, and interaction analysis.

[b] Reported type-I VEGFR2 inhibitor. [c] Reported type-II VEGFR2 inhibitor.

[*] Root mean square deviation (RMSD) was calculated by comparing the re-docked and crystallized conformations of N-cyclopropyl-6-[(6,7-dimethoxyquinolin-4-yl)oxy]naphthalene-1-carboxamide, a value of 0.85Å.

Supplementary Figure S6. (A) Molecular interaction of the human inactive site VEGFR2 (4ASD) with LA and reported inhibitors. (B) Molecular interaction of the human active site VEGFR2 (3B8R) with LA and reported inhibitors.
